# Poly(I:C) Potentiates T Cell Immunity to a Dendritic Cell Targeted HIV-Multiepitope Vaccine

**DOI:** 10.3389/fimmu.2019.00843

**Published:** 2019-04-24

**Authors:** Juliana de Souza Apostólico, Victória Alves Santos Lunardelli, Marcio Massao Yamamoto, Edecio Cunha-Neto, Silvia Beatriz Boscardin, Daniela Santoro Rosa

**Affiliations:** ^1^Laboratory of Experimental Vaccines, Department of Microbiology, Immunology and Parasitology, Federal University of São Paulo, São Paulo, Brazil; ^2^Institute for Investigation in Immunology (iii)—INCT, São Paulo, Brazil; ^3^Department of Parasitology, Institute of Biomedical Sciences, University of São Paulo, São Paulo, Brazil; ^4^Laboratory of Clinical Immunology and Allergy (LIM60), School of Medicine–University of São Paulo, São Paulo, Brazil

**Keywords:** HIV, multiepitope vaccine, dendritic cell targeting, DEC205, adjuvants

## Abstract

Cellular immune responses are implicated in resistance to HIV and have been considered for the development of an effective vaccine. Despite their safety profile, subunit vaccines need to be delivered combined with an adjuvant. In the last years, *in vivo* antigen targeting to dendritic cells (DCs) using chimeric monoclonal antibodies (mAb) against the DC endocytic receptor DEC205/CD205 was shown to support long-term T cell immunity. Here, we evaluated the ability of different adjuvants to modulate specific cellular immune response when eight CD4^+^ HIV-derived epitopes (HIVBr8) were targeted to DEC205^+^ DCs *in vivo*. Immunization with two doses of αDECHIVBr8 mAb along with poly(I:C) induced Th1 cytokine production and higher frequency of HIV-specific polyfunctional and long-lived T cells than MPL or CpG ODN-assisted immunization. Although each adjuvant elicited responses against the 8 epitopes present in the vaccine, the magnitude of the T cell response was higher in the presence of poly(I:C). Moreover, poly(I:C) up regulated the expression of costimulatory molecules in both cDC1 and cDC2 DCs subsets. In summary, the use of poly(I:C) in a vaccine formulation that targets multiple epitopes to the DEC205 receptor improved the potency and the quality of HIV-specific responses when compared to other vaccine-adjuvant formulations. This study highlights the importance of the rational selection of antigen/adjuvant combination to potentiate the desired immune responses.

## Introduction

Vaccine induced T cell immunity is required for effective protection against intracellular pathogens responsible for diseases classified as global threats like AIDS, tuberculosis, malaria, and also against cancer. The ability of dendritic cells (DCs) to uptake, process and present antigens is crucial to induce and regulate T cell immunity (1). Thus, activation of DCs has been considered key in vaccines designed to induce cellular immunity (2). DCs express a wide range of receptors including pattern recognition receptors (PRRs), like toll-like receptors (TLRs), cytosolic receptors, and C-type lectin receptors, that are able to recognize pathogen- or damage- associated molecular patterns (PAMPs or DAMPs, respectively) (3). The nature of the signal delivered to the DC does not only affect the magnitude of T cell responses, but also influences the generation of memory precursors and the overall quality of immune response (4, 5).

Human and mouse DCs can be divided in two major subsets: plasmacytoid DCs and conventional/myeloid DCs with specific functions in the steady state (6–8). Recently, DCs were classified based on their ontogeny in conventional type 1 DCs (cDC1) and conventional type 2 DCs (cDC2) (9, 10). Conventional type 1 DCs encompass lymphoid CD8α^+^ and non-lymphoid CD103^+^, both of which express DEC205. DEC205 also known as CD205 is a C-type lectin endocytic receptor and was the first identified DC-specific receptor (11). DEC205 is highly expressed on cDC1, but can also be found on thymic epithelial cells, Langerhans cells and, at relatively low levels, on B cells (12, 13). Recently, synthetic CpG oligonucleotides (ODNs), a potent immunostimulator, were identified as ligands that bind to the surface DEC205 (14, 15).

A promising strategy to improve vaccine efficacy is to selectively target the desired antigen to a DC subset by linking it to a monoclonal antibody (mAb) against the specific DC receptor. During the last decade, several reports revealed the feasibility of *in vivo* antigen targeting to cDC1 using a mAb against DEC205 (αDEC205) to improve both humoral and cellular responses (2, 16–20). Vaccination with DEC205 targeted antigens also induced protection in different infection and tumor models (21–23). However, for this particular receptor, inflammatory signals such as adjuvants must be co-administered with the targeted antigen to induce DC maturation, cellular immunity and avoid tolerance (24–26).

Different microbial products such as TLR ligands have been characterized and used as adjuvants to trigger intracellular signaling cascades that result in cytokine production, up regulation of costimulatory molecules and DCs maturation (27–30). Mouse conventional DC subsets differentially express a broad repertoire of TLRs that result in different activating phenotypes and adaptive immunity (31). The co-delivery of TLR ligands and DEC205 targeted antigens has been shown to significantly improve vaccine immunogenicity in mice and in non-human primates (16).

Polyinosinic:polycytidylic acid [poly(I:C)] is a synthetic analog of viral double-stranded RNAs (dsRNAs) that activates TLR3 and RIG-I-like receptors (retinoic acid-inducible gene -I- like receptors, or RLRs) (32). Poly(I:C) is the most commonly administered adjuvant in mice in the context of DC-targeted vaccines using αDEC205 mAbs fused with proteins of interest (18). This strategy has already been tested with chimeric mAbs containing proteins derived from dengue virus (33), *Trypanosoma cruzi* (34), *Plasmodium* sp (26, 35, 36), *Mycobacterium tuberculosis* (37), *Yersinia pestis* (22), *Toxoplasma gondii* (23), HIV (21, 38, 39) and also from tumors (40). The excellent results obtained with this adjuvant, justified its use in clinical trials. To improve poly I:C stability (32) in humans, a modified version (poly-ICLC) was developed and used in different trials (41, 42).

Monophosphoryl lipid A (MPL), a chemically derivative of bacterial lipopolysaccharide (LPS), is a TLR4 agonist that preferentially activates the TIR-domain-containing adapter-inducing interferon-β (TRIF) signaling pathway to drive the production of Th1 cytokines and activate CD4^+^ T cells (43) (44, 45). MPL is the first and only TLR ligand licensed in a human vaccine (Melacine™, approved as a melanoma vaccine). More recently, other MPL-containing vaccines became available (Fendrix™ and Cervarix™, both from GSK) (46). CpG oligodeoxynucleotides (ODN) are unmethylated CpG motifs that interact with endosomal TLR9 and lead to proinflammatory cytokine production by DCs (47). B type ODN has a protective phosphorothioate backbone that protects it from nuclease digestion and enhances its half-life *in vivo* (48). Several clinical trials were conducted and CpG ODN emerged as a potent adjuvant to induce high antibody titers more quickly and after fewer doses (49, 50). Moreover, CpG ODN has been used along with αDEC205 mAb to target HIV and *Plasmodium* proteins (51, 52).

Here, we used eight promiscuous HIV-derived CD4^+^ T cell epitopes (HIVBr8) fused with αDEC205 to target CD11c^+^ CD8α^+^ DCs in the presence of different TLR ligands. The hierarchy of adjuvant potency shows that poly(I:C) is a superior adjuvant for the multiepitope DC-targeted vaccine in magnitude, breadth, and longevity.

## Materials and Methods

### Generation of the Fusion Monoclonal Antibody (mAb)

Plasmids encoding the light and heavy chain of the mouse αDEC205 antibody were kindly provided by Dr. Michel C. Nussenzweig (The Rockefeller University, New York, USA). The plasmid encoding the heavy chain of the mouse DEC205 fused to eight HIV-1 epitopes was previously described and contains the following epitopes: p6 (32-46), p17 (73-89), pol (785-799), gp160 (188-201), rev (11-27), vpr (65-82), vif (144-158), and nef (180-194) (39).

### Expression and Purification of αDECHIVBr8 mAb

The production of αDECHIVBr8 mAb [original clone NLDC145 (24)] was performed after transient transfection of human embryonic kidney (HEK) 293T cells (ATCC, CRL-11268) exactly as described elsewhere (33). Briefly, 293T cells were cultured in 150 mm plates (Sarstedt) under standard conditions in Dulbecco's modified Eagle's medium (Invitrogen) supplemented with 1% (v/v) antibiotic-antimycotic (Invitrogen), 1% (v/v) l-glutamine (Invitrogen), and 5% (v/v) Ultra low IgG Fetal Bovine Serum (Invitrogen). When cell confluence reached 70%, 293T cells were transfected using 10 μg of the plasmids encoding the light and the heavy chains in the presence of 150 mM NaCl and 0.45 mg/mL polyethyleneimine (PEI) (Sigma Aldrich). After 7 days in culture at 37°C with 5% CO_2_, the culture supernatants containing secreted antibodies were collected by centrifugation at 1,000 x g for 30 min at 4°C and filtered through 0.22 μM membrane. The chimeric αDECHIVBr8 mAb was precipitated by addition of ammonium sulfate (Sigma Aldrich) to 60% of the total culture volume, and resuspended/dialyzed overnight against PBS at 4°C. After purification by affinity chromatography with protein G beads column (GE Healthcare), fusion mAb was dialyzed against PBS, resolved on a SDS-12% polyacrylamide gel, quantified, and stored at −20°C until use.

### Mice

Female BALB/c (H-2^d^) mice with 6-to 8-week old were purchased from Centro de Desenvolvimento de Modelos Experimentais para Medicina e Biologia (CEDEME)- Brazil. Mice were housed and manipulated under specific-pathogen-free (SPF) conditions at the animal care facility of the Division of Immunology, Federal University of São Paulo (UNIFESP).

### Immunization

Groups of six mice were immunized twice, 2 weeks apart, with 4 μg of αDECHIVBr8 mAb by intraperitoneal (I.P) route in the presence of the following adjuvants: 50 μg of poly(I:C) (Invivogen), 20 μg of Monophosphoryl Lipid A (MPL) (Invivogen), or 10 μg of CpG ODN 1826 (Invivogen). The amount of adjuvants used was previously determined (53). Control groups were immunized with 4 μg of αDECHIVBr8 in the absence of adjuvant or with PBS only.

### Spleen and Mesenteric Lymph Node Cell Isolation

Fifteen and sixty days after the administration of the second dose (boost), mice were deeply anesthetized by ketamine/xylazine solution (300 and 30 mg/kg, respectively) and mesenteric lymph nodes and the spleen were aseptically removed. After obtaining single cell suspensions, cells were washed in 10 mL of RPMI 1640 (Gibco). Splenic red blood cells were lysed with 1 mL of ACK solution (150 mM NH_3_Cl, 10 mM KHCO_3_, 0.1 mM EDTA) for 2 min at room temperature. After two additional washes with RPMI 1640, splenocytes and lymph node cells were then resuspended in R10 (RPMI supplemented with 10% of fetal bovine serum, 2 mM L-glutamine, 1% v/v vitamin solution, 1mM sodium pyruvate, 1% v/v non-essential amino acids solution, 40 μg/mL of Gentamicin, 5 x 10^−5^ M 2-mercaptoetanol (all from Gibco) and 20 μg/mL of Cyprofloxacin (Ciprobacter, Isofarma). The viability of cells was evaluated using 0.2% Trypan Blue exclusion dye to discriminate between live and dead cells. Cell concentration was estimated with the aid of a cell counter (Countess, Invitrogen) and adjusted in cell culture medium.

### Cytokine Determination

One million splenocytes were incubated for 48 h in the presence of pooled HIV-1 peptides (5 μM) or medium alone as negative control. Culture supernatants were harvested and stored at −20°C until analysis. IL-2, IL-4, IL-6, IL-10, IL-17, IFNγ, and TNFα were detected simultaneously using mouse Th1/Th2/Th17 cytokine bead array (CBA) kit (BD Pharmingen), according to the manufacturer's instructions. The range of detection was 20–5,000 pg/mL for each cytokine.

### T Cell ELISpot Assay

The ELISpot assay was performed using mouse IFNγ ELISpot Ready-SET-Go! (eBiosciences) according to manufacturer's instructions. Splenocytes from immunized mice were obtained as described and assayed for their ability to secrete IFNγ after *in vitro* stimulation with individual or pooled HIV-1 peptides (5 μM) or medium alone as negative control. Spots were counted using an AID ELISPOT Reader System (Autoimmun Diagnostika GmbH, Germany). The number of IFN-γ producing cells/10^6^ splenocytes was calculated after subtracting the negative control values and the cutoff was 15 SFU per million splenocytes.

### Analysis of HIV-Specific Proliferation and Intracellular Cytokine Production by Flow Cytometry

To analyze HIV-specific T cell expansion, proliferation, and cytokine production, splenocytes from immunized mice were labeled with carboxyfluorescein succinimidyl ester (CFSE) (54). In summary, freshly isolated splenocytes were resuspended (50 × 10^6^/mL) in PBS and labeled with 1.25 μM of CFSE (Molecular Probes) at 37°C for 10 min. The reaction was quenched with RPMI 1640 supplemented with 10% FBS (R10) and cells were washed/resuspended with R10. Cells were cultured in 96-well round-bottomed plates (5 × 10^5^/well in triplicate) for 5 days at 37°C and 5% CO_2_ with medium alone or with pooled HIV-1 peptides (5 μM). After 4 days, cells were restimulated with pooled HIV-1 peptides (5 μM) in the presence of 2 μg/mL anti-CD28 (BD Pharmingen) and Brefeldin A- GolgiPlugTM (BD Pharmingen) for further 12 h. After the incubation period, cells were washed with FACS buffer (PBS with 0.5% BSA and 2 mM EDTA) and surface stained with anti-mouse CD3 APCCy7 (clone 145-2C11), CD4 PerCP (clone RM4-5), and CD8 Pacific Blue (clone 53-6.7) monoclonal antibodies for 30 min at 4°C. Cells were fixed and permeabilized using Cytofix/Cytoperm™ kit (BD Pharmingen), according to manufacturer's instructions. After permeabilization, cells were washed with Perm/Wash buffer (BD Biosciences) and stained with anti-mouse IL2 PE (clone JES6-5H4), TNFα PECy7 (clone MP6-XT22), and IFNγ APC (clone XMG1.2) monoclonal antibodies for 30 min at 4°C. Following staining, cells were washed twice and resuspended in FACS buffer. All antibodies were from BD Pharmingen. Samples were acquired on a FACSCanto II flow cytometer (BD Biosciences) and then analyzed using FlowJo software (version 9.9, Tree Star, San Carlo, CA). To analyze cellular polyfunctionality, we used the Boolean gate platform (FlowJo software) to create combinations of the three cytokines (IL-2, TNFα, and IFNγ) within the CFSE^low^ population (cells that have undergone at least one cycle of division) resulting in seven distinct patterns. Polyfunctionality was defined as the ability of cells to exert at least two functions. The gating strategy, illustrated using data from one representative experiment, is shown in Figure S1. The frequencies of cytokine producing cells were calculated by subtracting the frequency of cells that were stimulated *in vitro* with HIV peptides by the frequency of the cells that were cultured in the presence of medium alone (background). For each experiment performed, unstained and all single-color controls were processed to allow proper compensation.

### Expression of Costimulatory Molecules on DC Surface

Mice were immunized once with 4 μg of αDECHIVBr8 mAb combined with the different adjuvants (poly(I:C), MPL or CpG ODN 1826). After 12 h, splenocytes were stained with biotinylated anti-mouse CD3 (clone 145-2C11), CD19 (clone 1D3), and CD49b (clone DX5). After 30 min, cells were washed with FACS buffer and stained with streptavidin APCCy7, anti-mouse CD11c APC (clone HL3), IAIE PE (clone 2G9), CD8 Pacific Blue (clone 53-6.7), CD40 FITC (clone 3.23), CD80 PerCP (clone 16-10A1), and CD86 PECy7 (clone GL1). Samples were acquired on a FACSCanto II flow cytometer (BD Biosciences) and then analyzed using FlowJo software (version 9.9, Tree Star, San Carlo, CA). For each experiment performed, unstained and all single-color controls were processed to allow proper compensation. Three million events were acquired in a live lymphocyte gate.

### Data Analysis

Statistical significance (*p*-value) was calculated by Two-way ANOVA followed by Bonferroni *post hoc* test or unpaired *t*-test (different time points comparison). Statistical analysis and graphical representation of data was performed using GraphPad Prism version 7.0 software.

## Results

### Multiepitope Targeting to DEC205^+^ DCs With Different Adjuvants Induces Type 1 Cytokine Production

**Figure 1:**
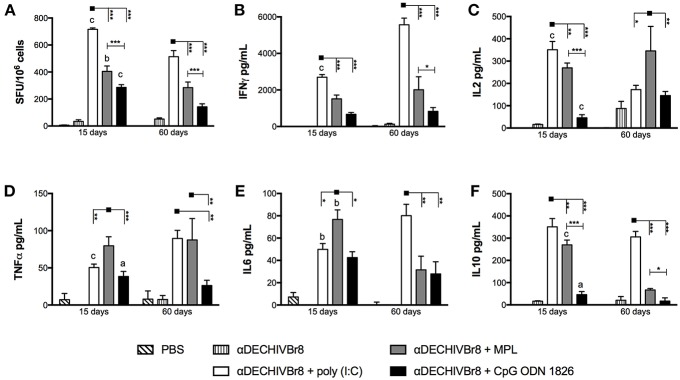
Properties of HIV-specific cellular imune response after immunization with multiepitope αDECHIVBr8 mAb in the presence of adjuvants. BALB/c mice (*n* = 6) were immunized with two doses of 4 μg of αDECHIVBr8 along with poly(I:C), MPL or CpG ODN 1826. Control groups received αDECHIVBr8 only or PBS. Fifteen and sixty days after the boost the splenocytes were **(A)** cultured in the presence of pooled HIV-1 peptides (5 μM) for 18 h to evaluate the number of IFN-γ producing cells by ELISpot assay. SFU, spot forming units. Cutoff = 15 SFU/10^6^ cells and is represented by the dotted line. **(B–F)** cultured in the presence of pooled HIV-1 peptides for 48 h to measure IFNγ **(B)**, IL2 **(C)**, TNFα **(D)**, IL6 **(E)**, and IL10 **(F)** in culture supernatants by flow cytometry. Data were analyzed by two-way ANOVA followed by Bonferroni *post hoc* test or unpaired *t-test* (different time points comparison) ^*^p < 0.05, ^**^p < 0.01; ^***^p < 0.001; a *p* < 0.05; b *p* < 0.01; c *p* < 0.001 when 15 days was compared to 60 days time point. Data represent mean ± SD and are representative of 3 independent experiments.

To examine the effect of different adjuvants on HIV-specific cellular immune response, mice were immunized with two doses of αDECHIVBr8 mAb in the presence of the TLR agonists poly(I:C), MPL or CpG ODN 1826. Fifteen or Sixty days after the boost, splenocytes from immunized mice were incubated with pooled HIV-1 peptides to analyze specific cytokine production. First, we evaluated IFNγ production by ELISpot assay (Figure 1A). We observed that 15 days after the boost splenocytes from mice immunized with αDECHIVBr8 mAb combined with poly(I:C) presented higher number of specific IFNγ producing cells (716 SFU/10^6^ cells) when compared to the groups immunized in the presence of MPL or CpG ODN 1826 (404 and 286 SFU/10^6^ cells, respectively). Moreover, a significant difference was observed between MPL and CpG ODN 1826 groups (Figure 1A, left). Sixty days after the boost, we detected the same profile albeit with lower magnitude. Mice immunized with αDECHIVBr8 combined with poly(I:C) displayed 514 SFU/10^6^ cells while MPL and CpG ODN 1826 presented 284 and 142 SFU/10^6^ cells, respectively (Figure 1A, right). A comparison between 15 and 60 days revealed a significant decrease in the magnitude for poly(I:C) (*p* < 0.001), CpG ODN 1826 (*p* < 0.001), and MPL (*p* < 0.01) immunized groups. Splenocytes from mice immunized with αDECHIVBr8 in the absence of adjuvant or PBS (control groups) presented negligible numbers of IFNγ producing cells.

We also analyzed the cytokine profile by CBA assay using supernatant culture of splenocytes stimulated with pooled HIV peptides. Splenocytes from mice that received αDECHIVBr8 combined with poly(I:C) produced higher levels of IFNγ when compared to MPL or CpG ODN 1826, corroborating the ELISpot findings (Figure 1B). Interestingly, in the poly(I:C) adjuvanted group IFNγ production was even higher 60 days after the boost when compared to the 15 days time point (*p* < 0.001). Poly(I:C) also induced superior IL-2 production 15 days after the boost (Figure 1C, left). However, 60 days after the boost, IL-2 production significantly decreased in the group immunized with αDECHIVBr8 plus poly(I:C) and increased in the group that received the mAb in the presence of MPL (*p* < 0.001) (Figure 1C, right). IL-2 production by the group that received the mAb with CpG ODN 1826 slightly increased 60 days after the boost when compared to 15 days time point (*p* < 0.001). Regarding TNFα production 15 days after the boost, we observed that αDECHIVBr8 mixed with MPL produced the highest levels (Figure 1D). TNFα levels increased 60 days after the boost for the poly(I:C) group (*p* < 0.001) and decreased for the CpG ODN 1826 group (*p* < 0.05). No difference was observed for the MPL immunized group. Inflammatory IL-6 (Figure 1E) was higher in the group immunized with αDECHIVBr8 plus MPL 15 days after boost, but at the later time point the levels of this cytokine significantly decreased (*p* < 0.01). In contrast, 60 days after the boost with mAb and poly(I:C), IL-6 (*p* < 0.01) production increased considerably. IL-10 (Figure 1F) was superior in the poly(I:C) immunized group in both time points followed by MPL immunized group. However, after 60 days, IL-10 production decreased in the MPL (*p* < 0.001) and in the CpG ODN 1826 (*p* < 0.05) groups. Of note, IL-4 and IL-17 production was below the assay detection limit (data not shown). Taken together, these results indicate that different adjuvants induce a type 1 immune response when multiple HIV-antigens are delivered to CD8α^+^ DCs by the endocytic receptor DEC205.

### Poly(I:C) Promotes Robust and Long-Lived Polyfunctional T Cell Responses

**Figure 2:**
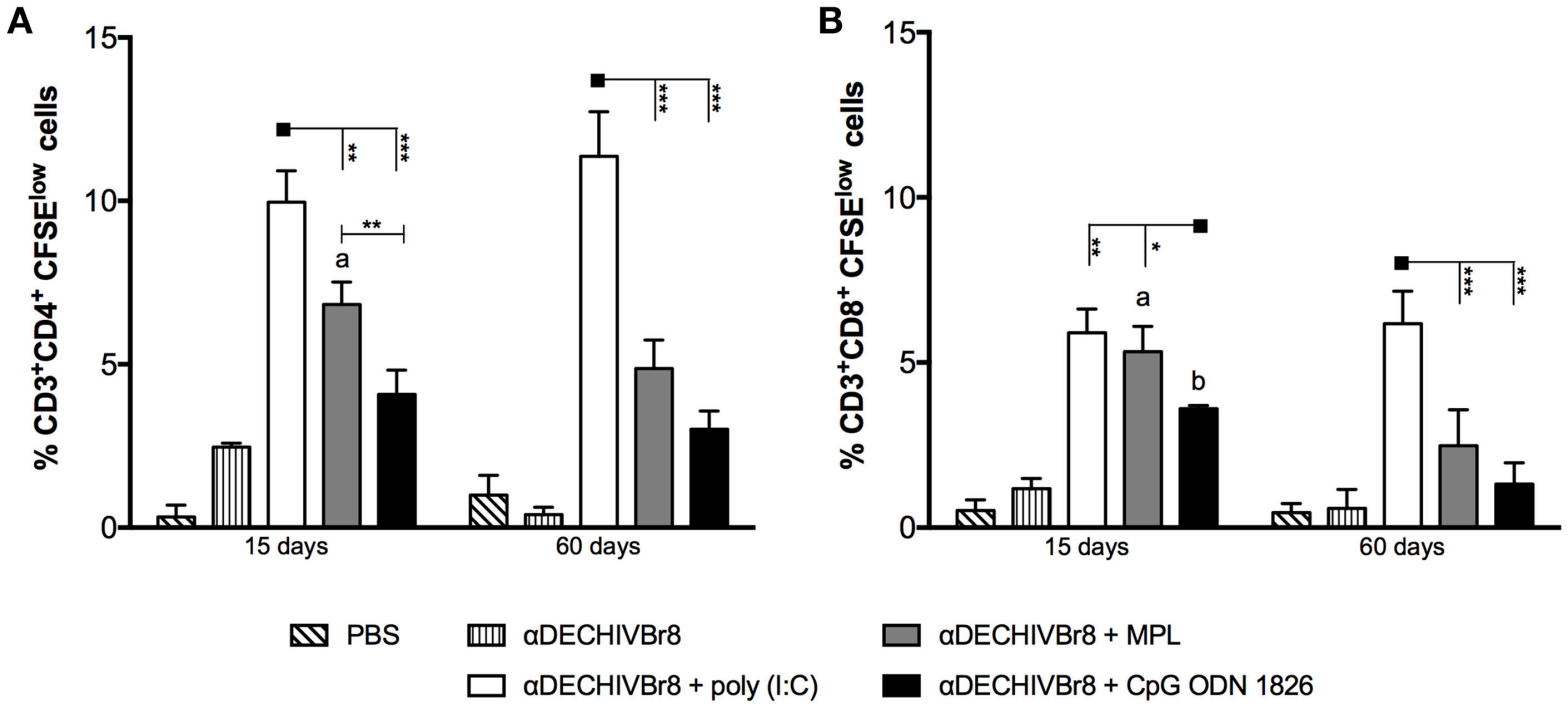
Immunization with αDECHIVBr8 mAb in the presence of poly(I:C) induces robust HIV-specific T cell proliferation. BALB/c mice (*n* = 6) were immunized as in Figure 1. Fifteen and 60 days after the boost the splenocytes were labeled with CFSE and cultured in the presence of pooled HIV-1 peptides (5 μM) for 5 days to evaluate specific proliferation. CFSE dilution on gated **(A)** CD3^+^CD4^+^ or **(B)** CD3^+^CD8^+^ cells was used as readout for antigen-specific proliferation. One million events were acquired in a live lymphocyte gate. Data were analyzed by two-way ANOVA followed by Bonferroni *post hoc* test or unpaired *t-test* (time points comparison). ^*^p < 0.05, ^**^p < 0.01; ^***^p < 0.001. a *p* < 0.05; b *p* < 0.01; c *p* < 0.001 when 15 days was compared to 60 days time points. Data represent mean ± SD and are representative of 3 independent experiments.

**Figure 3:**
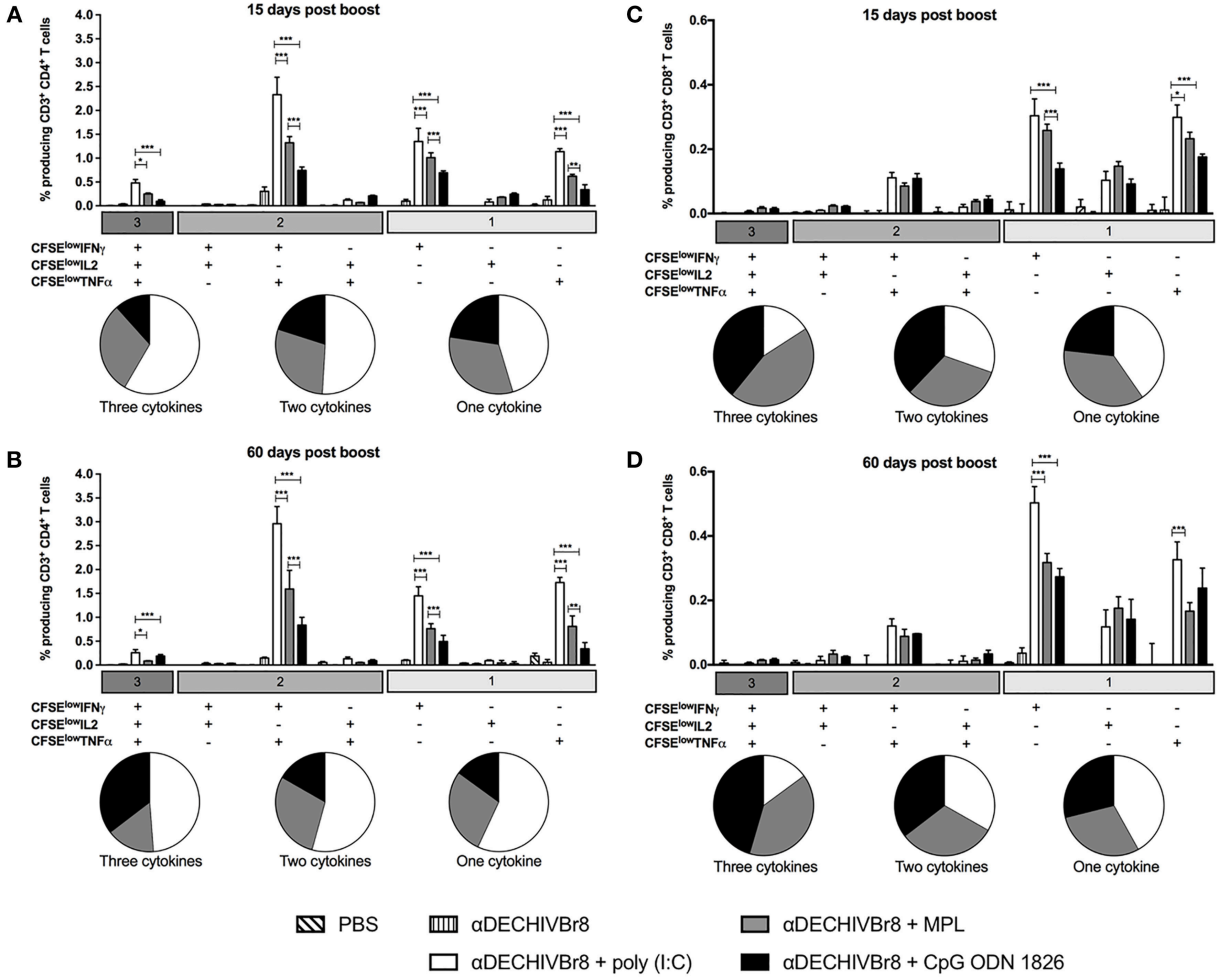
Poly(I:C) induces superior polyfunctional and long-lived HIV- specific T cell responses. BALB/c mice (*n* = 6) were immunized as in Figure 1. The splenocytes were labeled with CFSE and cultured in the presence of pooled HIV-1 peptides (5 μM) to evaluate specific proliferation and cytokine production by multiparameter flow cytometry. After gating on proliferating (CFSE^low^) and cytokine-producing cells, boolean combinations were created using FlowJo software to determine the frequency of each response based on all possible combinations of cytokine-producing CD4^+^ T cells **(A,B)** and CD8^+^ T cells **(C,D)** 15 and 60 days after the boost. Pie charts represent the proportion of T cells producing 1, 2, or all 3 cytokines. One million events were acquired in a live lymphocyte gate. Data were analyzed by two-way ANOVA followed by Bonferroni *post hoc* test. ^*^p < 0.05, ^**^p < 0.01; ^***^p < 0.001. Data represent mean ± SD and are representative of 3 independent experiments.

In an attempt to evaluate HIV-specific CD4^+^ and CD8^+^ T cell responses, splenocytes from immunized mice were labeled with CFSE and pulsed *in vitro* with HIV-1 peptides. After culture, the frequency of CD3^+^CD4^+^CFSE^low^ (Figure 2A) and CD3^+^CD8^+^CFSE^low^ (Figure 2B) were evaluated by flow cytometry. Fifteen days after boost, splenocytes from mice that received αDECHIVBr8 along with poly(I:C) presented higher frequency of proliferating CD4^+^ (9.96%) and CD8^+^ (5.90%) T cells when compared to MPL immunized groups (6.83 and 4.86%, respectively). In contrast, CpG ODN 1826 displayed the lowest frequency of proliferating T cells. The same profile was observed 60 days after the boost, with the group that received αDECHIVBr8 plus poly(I:C) displaying higher CD4^+^ (11.30%) and CD8^+^ (6.17%) specific proliferation when compared to MPL (CD4^+^CFSE^low^ 4.86% and CD8^+^CFSE^low^ 2.47%) or CpG ODN 1826 (CD4^+^CFSE^low^ 3.60% and CD8^+^CFSE^low^ 1.31%) (Figures 2A,B right, respectively). Comparative analyses showed significant difference on the frequency of CD4^+^CFSE^low^ cells between 15 and 60 days only for the group that received αDECHIVBr8 plus MPL (*p* < 0.05). Regarding the CD8^+^ T cell compartment (CD8^+^CFSE^low^ cells), a significant difference was observed for MPL (*p* < 0.05) or CpG ODN 1826 (*p* < 0.01) groups. In contrast, mice immunized with αDECHIVBr8 in the presence of poly(I:C) displayed similar frequency of proliferating CD4^+^ and CD8^+^ T cells in all time points. To further characterize the functional profile of antigen-specific T cells, we assessed the ability of single cells to proliferate and produce the cytokines IFNγ, TNFα, and IL2 individually or simultaneously. The flow cytometry profile demonstrated that immunization with αDECHIVBr8 mAb along with poly(I:C) induced higher frequency of CD4^+^ T cells that proliferated and produced IFNγ^+^IL2^+^TNFα^+^ or IFNγ^+^TNFα^+^ simultaneously or only one cytokine (IFNγ or TNFα) 15 or 60 days after the boost (Figures 3A,B, respectively). Interestingly, for the poly(I:C) and MPL groups 60 days after the boost, the frequency of polyfunctional CD4^+^ T cells that proliferated and produced IFNγ, TNFα, and IL-2 simultaneously decreased, leading to an increase in the double or single cytokine producers (Figure 3–pie charts). Moreover, αDECHIVBr8 mixed with poly(I:C) also displayed higher frequency of proliferating CD8^+^ T cells that produce IFNγ or TNFα 15 or 60 days after the boost when compared with other groups (Figures 3C,D, respectively). Similarly to what was observed with the CD4 compartment at the later time point (60 days), there was also a shift in the CD8^+^ T cell polyfunctional profile in all groups when compared to 15 days after the boost; the frequency of three cytokine producing cells diminished while the single cytokine producers augmented (Figure 3 pie charts). Altogether, these results demonstrated that immunization with two doses of αDECHIVBr8 along with poly(I:C) induced higher and long-lasting specific polyfunctional CD4^+^ and CD8^+^ T cells responses.

### Poly(I:C) Increases Epitope Coverage

**Figure 4:**
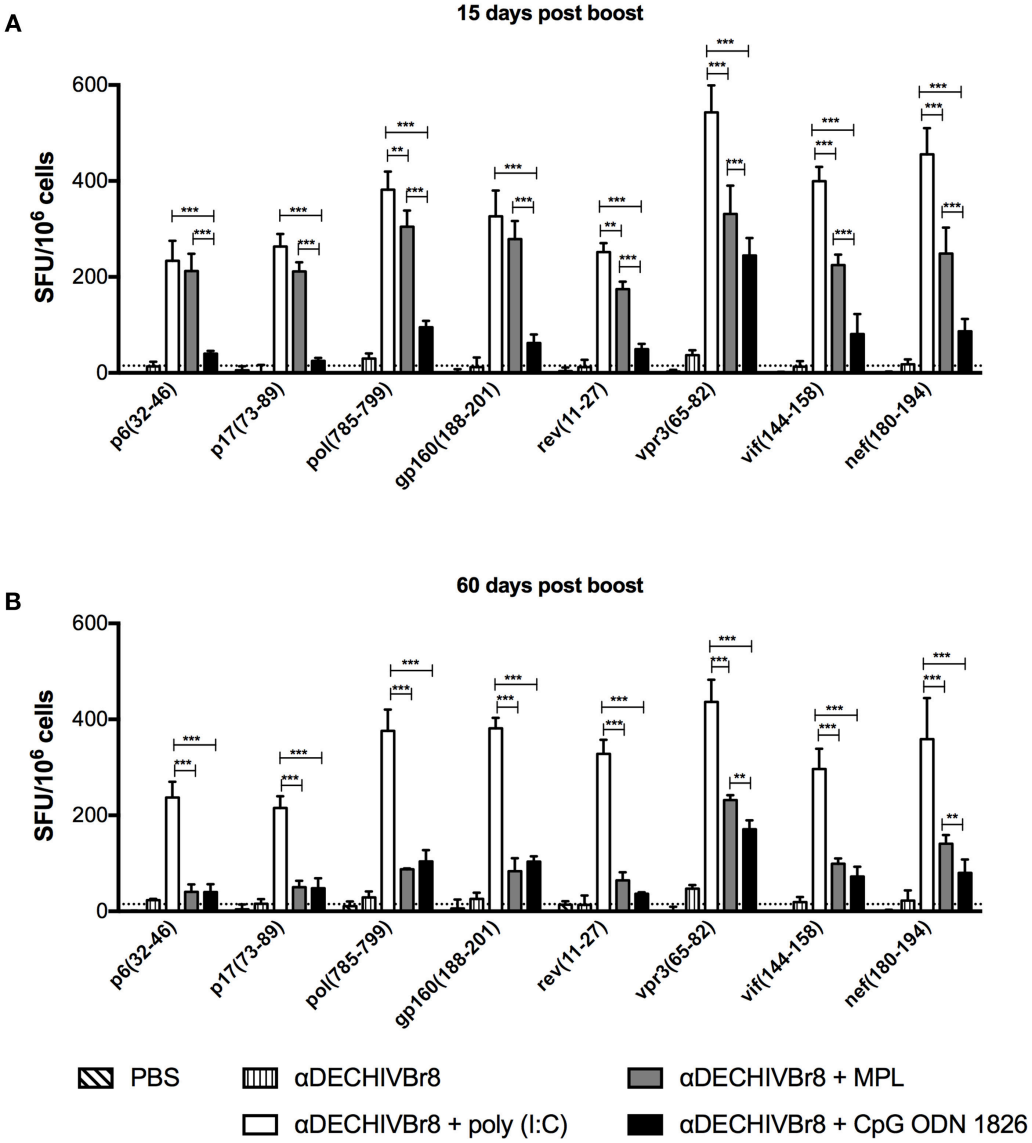
Multiepitope DC targeting in the presence poly(I:C) induces broad T cell responses. BALB/c mice (*n* = 6) were immunized as described in Figure 1. **(A)** Fifteen and **(B)** 60 days after the boost the splenocytes were cultured with single HIV-1 peptides (5 μM) for 18 h to evaluate the number of IFN-γ producing cells by ELISpot assay. SFU, spot forming units. Cutoff = 15 SFU/10^6^ cells and is represented by the dotted line. Data were analyzed by two-way ANOVA followed by Bonferroni *post hoc* test. ^**^p < 0.01; ^***^p < 0.001. Data represent mean ± SD and are representative of 4 independent experiments.

To assess the breadth of T cell responses, splenocytes from immunized mice were incubated with single HIV-1 peptides present in the fusion vaccine and the number of IFNγ producing cells was determined by ELISpot. Fifteen days after last dose (Figure 4A), all adjuvants tested were able to induce positive responses against all peptides, albeit at different magnitudes (poly(I:C) > MPL > CpG ODN). At a later time point (Figure 4B), poly(I:C), and CpG ODN adjuvanted groups sustained IFNγ production against all peptides (head-to-head comparison in Figures S2A,C). On the contrary, in the MPL group, the magnitude of the response was more significantly reduced when we compared the 15 and 60 days time points (Figure S2B). Thus, multiepitope *in vivo* targeting to DEC205^+^ DCs when combined with poly(I:C) induced broad, potent and long-lasting T cell responses.

### Differential Expression of Costimulatory Molecules in Splenic DCs Subsets

**Figure 5:**
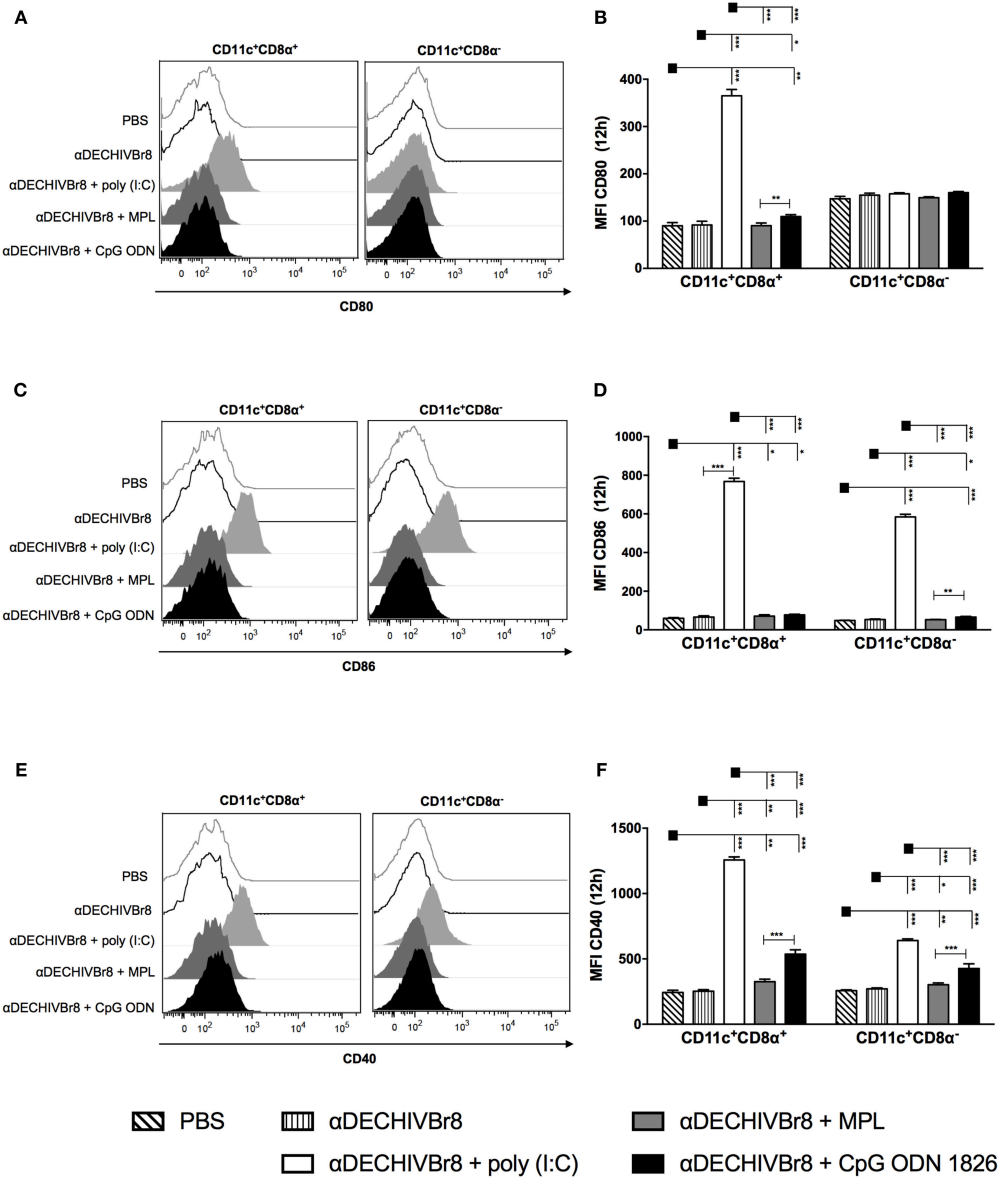
DC targeting in the presence of different adjuvants modulates the expression of costimulatory molecules in splenic DC subsets. BALB/c mice (*n* = 5) were injected with 4 μg of αDECHIVBr8 plus poly(I:C), MPL or CpG ODN 1826. Control groups received αDECHIVBr8 only or PBS. After 12 h the splenocytes were labeled with fluorescent antibodies and 3 million events were acquired. Initial gating included a single cell gate followed by selection of CD3^−^CD19^−^CD49b^−^ population. DCs were identified as CD11c^+^ IAIE ^+^, subsequently gated on CD8α^+^ and CD8α^−^ and the median fluorescence intensity (MFI) of CD80 **(A,B)**, CD86 **(C,D)**, and CD40 **(E,F)** was determined in each DC subset. Data were analyzed by two-way ANOVA followed by Bonferroni *post hoc* test. ^*^p < 0.05, ^**^p < 0.01; ^***^p < 0.001. Data represent mean ± SD and are representative of 3 independent experiments.

To further characterize phenotypic differences among the adjuvants, we compared the maturation status of splenic DCs after *in vivo* administration of the mAb combined with poly(I:C), MPL or CpG ODN 1826. The gating strategy, illustrated using data from one representative experiment, is shown in Figure S3. Twelve hours after injection, CD11c^+^CD8α^+^ DCs from poly(I:C) group considerably up-regulated the expression of CD80 compared to other groups (Figures 5A,B). CpG ODN 1826 slightly increased CD80 expression only when compared to MPL. However, none of the adjuvants up regulated CD80 expression on CD11c^+^CD8α^−^ DCs. Furthermore, poly(I:C) was the only adjuvant to significantly up regulate CD86 expression in both DCs subsets (Figures 5C,D). Similarly, we observed a significant increase in the MFI of CD40 molecule by poly(I:C) in both DCs subsets when compared to other adjuvants (Figures 5E,F). In addition, to assess whether DC activation could occur earlier than 12 h, we analyzed the expression of costimulatory molecules 6 h after injection, and observed the same pattern of CD80, CD86, and CD40 expression in both DCs subsets (Figures S4A–C, respectively). We also analyzed the activation profile on mesenteric lymph nodes and the same pattern of expression was observed (data not shown). Taken together, these results strength the idea that poly(I:C) is a superior adjuvant than MPL or CpG ODN 1826 since it up regulates costimulatory molecules in both splenic DCs subsets (CD8α^+^ and CD8α^−^).

## Discussion

Antigen targeting to DCs through DEC205 endocytic receptor is an effective way to enhance antigen uptake. However, the induction of cell immunity is only accomplished when αDEC205 chimeric mAbs are delivered together with an adjuvant (55–57). Adjuvants enhance immunity to vaccine antigens by influencing the magnitude, breadth/immunodominance, and persistence of immune responses (27). Hence, the choice of the adjuvant formulation is of utmost importance to induce the desired immune response (58). Although a limited number of vaccine adjuvants are currently licensed for human use (aluminum salts, MF59, AS03, and AS04), several compounds have entered clinical trials with demonstrated efficacy (27).

Antigen targeting to DCs through DEC205 receptor is used as a vaccination strategy to induce strong antigen-specific immune responses against several pathogens (26, 34, 55) and tumors In the HIV vaccine scenario, antigen targeting to cDC1 through DEC205 was performed using the full-length gag (p24) protein (21, 51, 59–62). The success in different pre-clinical studies using mice and non-human primates (16) quickly pushed forward the translation of this strategy to humans. Recently, two phase I clinical trials (NCT01889719 and NCT01127464) delivered HIV p24 using a human αDEC205 mAb plus poly-ICLC as adjuvant. Promising results were obtained when a human αDEC205 mAb fused to the full-length tumor antigen NY-ESO-1 was administered together with poly-ICLC (41, 42). In fact, three phase I/IIb clinical trials are currently under way (NCT02166905, NCT03206047, NCT03358719) and two others are already completed (NCT01522820, NCT00948961, NCT01834248).

Previously, we generated an αDEC205 multiepitope fusion mAb (αDECHIVBr8) to target eight promiscuous CD4^+^ T cell epitopes from several HIV proteins to cDC1s. The αDECHIVBr8 mAb was administered to mice in the presence of poly(I:C) as adjuvant and compared to DNA plasmid immunization in homologous and heterologous prime-boost regimens. We found that αDECHIVBr8 homologous prime-boost regimen induced stronger T cell immune responses against all epitopes when compared to homologous DNA vaccination (39). Here, we compared the adjuvant properties of poly(I:C), MPL, and CpG ODN 1826 to induce HIV-specific cellular immune response when formulated with the fusion αDECHIVBr8 mAb. To our knowledge, this is the first time that multiple epitopes derived from different proteins of the same pathogen are targeted *in vivo* to DCs and tested in the context of different adjuvants. This is an important issue since adjuvants can influence immunodominance by altering the immune repertoire of CD4 T cell responses (63). Overall, our data reveal the potential of poly(I:C) as a superior adjuvant for the development of a multiepitope-based vaccine that targets CD8α^+^ DCs through the DEC205 endocytic receptor.

Initially, we found that poly(I:C) induced higher magnitude of specific IFNγ producing cells and also Th1 cytokine production when compared to MPL or CpG ODN 1826. Likewise, Longhi et al. showed that poly(I:C) is a more potent adjuvant to induce specific immune responses against a DC-targeted HIV gag protein (51). Indeed, poly(I:C) has been the most commonly administered adjuvant with DC-targeted vaccines using αDEC205 mAbs fused with full-length proteins from different pathogens in both mice and non-human primates (21–23, 33–39, 60).

Poly(I:C) is sensed by TLR3 and RLR receptors, and triggers up regulation of costimulatory molecules, strong type I IFN production by DCs and Th1 responses (32). Type I IFNs mediate the adjuvant effect of poly(I:C) acting as a third signal by promoting and sustaining clonal expansion of T cells (64–68). Indeed, our results demonstrate that immunization with αDECHIVBr8 along with poly(I:C) also induced higher frequency of proliferating CD4^+^ and CD8^+^ T cells. Moreover, we found that administration of the αDECHIVBr8 mAb concomitant with poly(I:C) induced higher frequency of specific polyfunctional T cells, i.e., cells that proliferated and simultaneously produced Th1 cytokines (IFNγ, IL2, and TNFα). Ours results corroborate with previous reports showing the development of polyfunctional T cells after HIV gag protein targeting to DCs along with poly(I:C) (21, 38, 62). Additionally, the presence of polyfunctional T cells is also a hallmark after vaccinia and yellow fever virus vaccinations (69, 70), and correlates with non-progressive HIV infection (71, 72). Recent HIV vaccine trials suggest that a broad (multiple specificities) and potent (high magnitude) response against conserved epitopes would be a desirable attribute of a T-cell based vaccine (73, 74). Indeed, vaccine induced broad T cell responses conferred protection after simian immunodeficiency virus challenge (75). We showed that poly(I:C) and MPL induced T cell responses against all epitopes (broad responses) present in the αDECHIVBr8 fusion mAb, although poly(I:C) was more potent. Likewise, Teixeira et al. demonstrated the ability of a bacterial adjuvant (*Propionibacterium acnes*) to expand the breath of a multiepitope DNA-based HIV vaccine (76).

A central feature of successful vaccines is their ability to induce immunological memory. Cross-sectional studies of smallpox and yellow fever vaccines showed that specific humoral and T cell responses can be detected for many years (77, 78). When we analyzed the longevity of the immune response, only poly(I:C) vaccine group had sustained T cell proliferation and IFNγ responses against all peptides ~2 months after the second immunization. It is important to note that MPL was the second most potent adjuvant tested and better to induce pro-inflammatory cytokines such as TNFα and IL-6. Previous reports provided evidence that MPL, a TLR2, and TLR4 agonist, is effective to induce TNFα, IL-10, and IL-12 production (44, 79). MPL induced a broad T cell response after the boost but narrowed after 2 months. Previous reports using αDEC205 mAb fused with HIV gag protein showed that MPL or LPS were as effective as poly(I:C) to induce specific humoral responses but less potent to induce Th1 CD4^+^ T cell immunity (38, 51).

Interestingly, immunization with αDECHIVBr8 in the presence of CpG ODN induced weak T cell responses and narrowed epitope positivity. B class CpG ODN is a fully phosphorothioate TLR9 agonist that binds to surface DEC205 receptor (14, 15) and could therefore compete with the fusion αDEC205 mAb for cellular uptake. Our data are in line with a previous study demonstrating that immunization with αDEC-Gag plus CpG ODN 1826 induces lower frequency of responding CD4^+^ T cells compared with poly(I:C) (51).

Anti-DECHIVBr8 combined with poly(I:C) was the most effective strategy to modulate DC activation by up regulating costimulatory molecules in a more pronounced way in the CD11c^+^ CD8α^+^ subset but also in CD11c^+^ CD8α^−^ DCs. This may be due to the fact that CD8α^+^ DEC205^+^ DCs express higher levels of TLR3 when compared to CD8α^−^ DCs (2, 18, 80). As a consequence of DC maturation, poly(I:C) enhanced T cell immunity. As stated before, it was shown that poly(I:C) was most effective to induce Th1 CD4^+^ T cell immunity compared to LPS or CpG ODN 1826 using the HIV gag targeted protein (51).

The use of mouse model to select an adjuvant may be a caveat since the pattern of expression of TLR in the target DEC205^+^ DC subset can differ between human and mouse (18). However, the adjuvant effect after antigen targeting does not necessarily rely on the direct activation of its respective TLR. For example, the effect of poly(I:C) on cDC1 is mediated by type I IFN receptor (51) suggesting that it is possible to have immune activation even if the targeted DC does not express a certain TLR.

Collectively, the observations demonstrate that combination of poly(I:C) with multiepitope targeting to DEC205^+^ DCs modulates DC activation and elicits strong, broad, polyfunctional, and long-lived Th1 responses superior to other adjuvants both in quantity and quality. Therefore, the pursuit of a safe and effective T cell-based vaccine may benefit from the proper association of multiple epitope targeting to DC populations using a potent adjuvant formulation.

## Ethics Statement

This study was carried out in accordance with the recommendations of the Federal Law 11.794 (2008) and the Guide for the Care and Use of Laboratory Animals of the Brazilian National Council of Animal Experimentation (CONCEA). The protocol was approved by the UNIFESP Animal Care and Use Committee (IACUC).

## Author Contributions

JA, SB, and DR conceived and designed the experiments. JA, VL, MY, and DR performed the experiments. JA, VL, and DR analyzed the data and prepared the figures. DR, SB, and EC-N contributed with reagents and materials. JA, VL, SB, and DR wrote the manuscript. SB, EC-N, and DR performed the final review of the article. All authors read and approved the final article.

### Conflict of Interest Statement

The authors declare that the research was conducted in the absence of any commercial or financial relationships that could be construed as a potential conflict of interest.
