# Supplementary material for: Poly(I:C) Potentiates T Cell Immunity to a Dendritic Cell Targeted HIV-Multiepitope Vaccine
Source: Front Immunol. 2019 Apr 24;10:843. doi: 10.3389/fimmu.2019.00843 (PMC6492566; doi:10.3389/fimmu.2019.00843)
Supplement: Supplementary file 1 [file Data_Sheet_1.docx]

Supplementary Material

Article Title

Juliana de Souza Apostólico, Victória Alves Santos Lunardelli, Marcio Massao Yamamoto, Edecio Cunha-Neto, Silvia Beatriz Boscardin, Daniela Santoro Rosa

*** Correspondence:** Daniela Santoro Rosa: dsantororosa@gmail.com


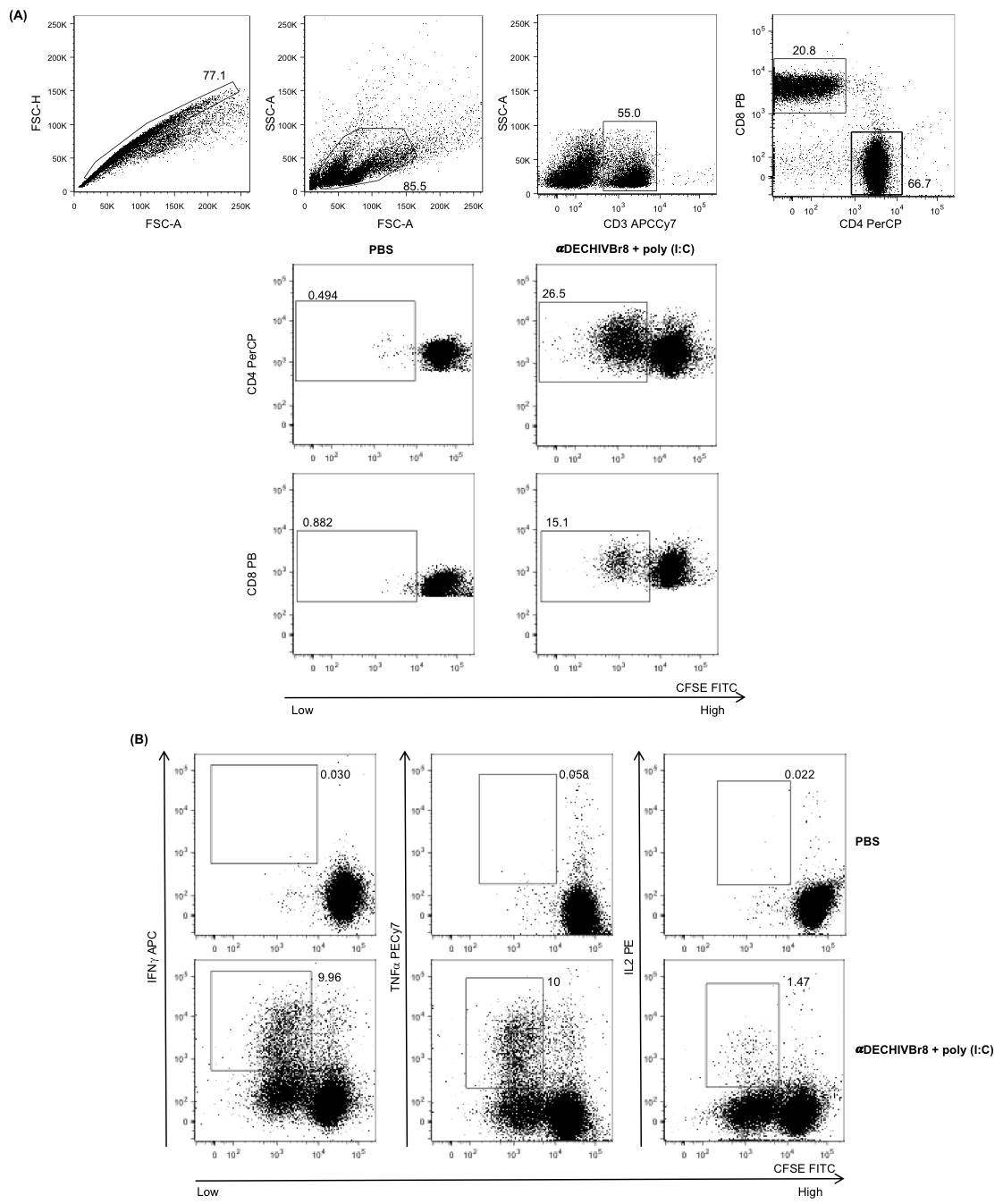


**Supplementary Figure 1. Flow cytometer analysis of cellular proliferation and intracellular cytokine production**. Representative dot plots of multiparameter flow cytometry after *in vitro* stimulation with pooled HIV peptides. **(A)** Gating strategy was first based on singlets, followed by lymphocytes (FSC vs SSC), CD3^+^ positive cells and CD4^+^ / CD8^+^ selection. Then, the frequency of proliferating (CFSE^low^) and cytokine-producing CD4^+^ and CD8^+^ T cells **(B)** was analyzed. Representative dot plots of PBS and αDEC205HIVBr8 + poly(I:C) immunized group are displayed.


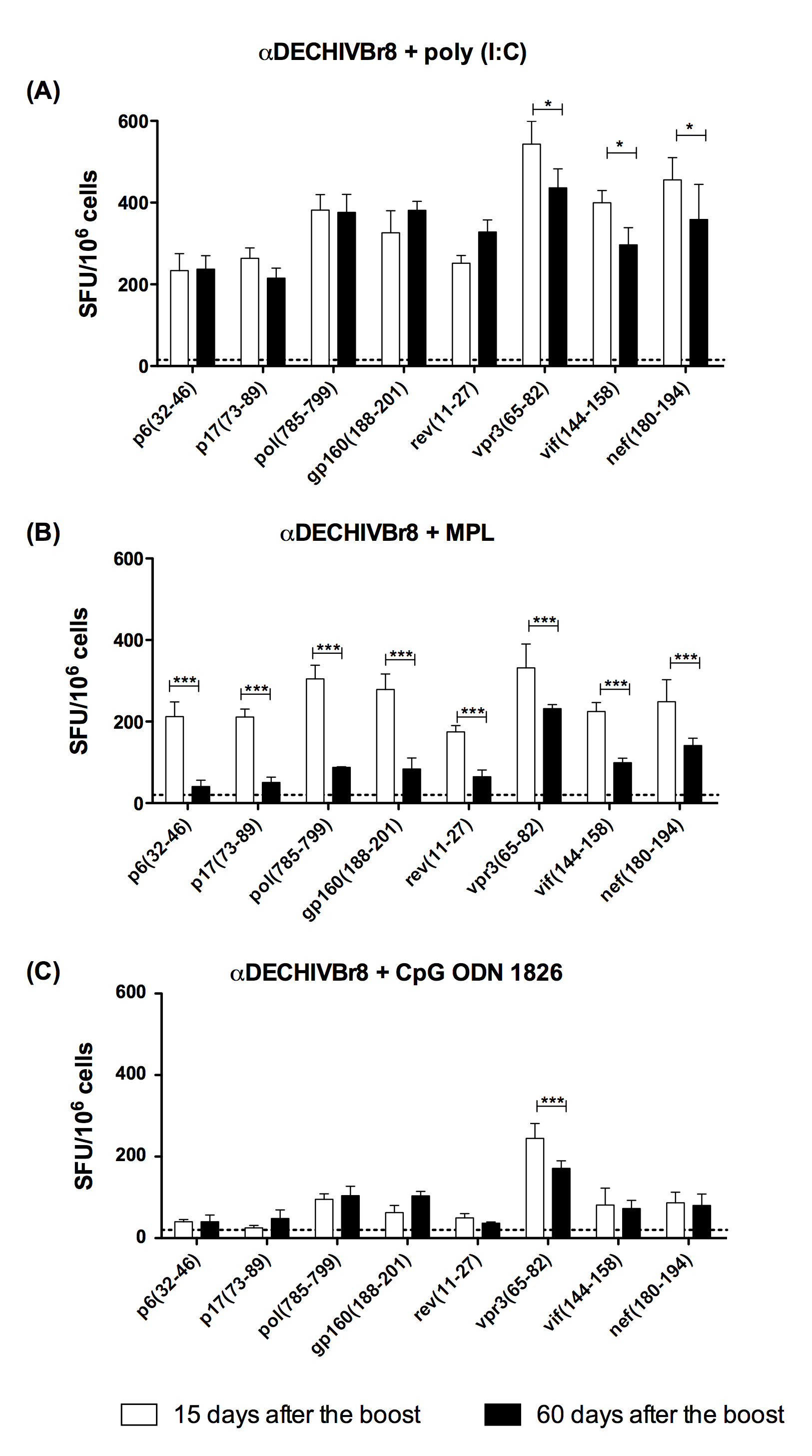


**Supplementary Figure 2**. **Multiepitope DC targeting in the presence poly(I:C) sustained broad and high magnitude of T cell responses.** BALB/c mice (n=6) were immunized with two doses of 4μg of αDECHIVBr8 along with (A) poly(I:C), (B) MPL or (C) CpG ODN 1826. Fifteen (white bars) and 60 days (black bars) after the boost the splenocytes were cultured with single HIV-1 peptides (5μM) for 18 hours to evaluate the number of IFN-γ producing cells by ELISpot assay. SFU: spot forming units. Cutoff = 15 SFU/10^6^ cells and is represented by the dotted line. Data were analyzed by two-way ANOVA followed by Bonferroni post hoc test.**p<0.01; ***p<0.001. Data represent mean ± SD and are representative of 4 independent experiments.

**Supplementary Figure 3.** **Gating strategy to analyze dendritic cell maturation.** BALB/c mice (n=5) were injected with 4μg of αDECHIVBr8 in the presence of poly (I:C), MPL or CpG ODN 1826. Control groups received αDECHIVBr8 only or PBS. After 12 hours the splenocytes were labeled with fluorescent antibodies and three million events were acquired. Initial gating included a single cell gate (based on height (FSC-H) and area (FSC-A)) followed by selection of CD3^-^CD19^-^CD49b^-^ population. DCs were identified as CD11c^+^ IAIE ^+^ and subsequently gated on CD8α^+^ (cDC1) and CD8α^-^ (cDC2). The median fluorescence intensity (MFI) of CD80, CD86 and CD40 was determined in each DC subset. Data are representative of 3 independent experiments.

**
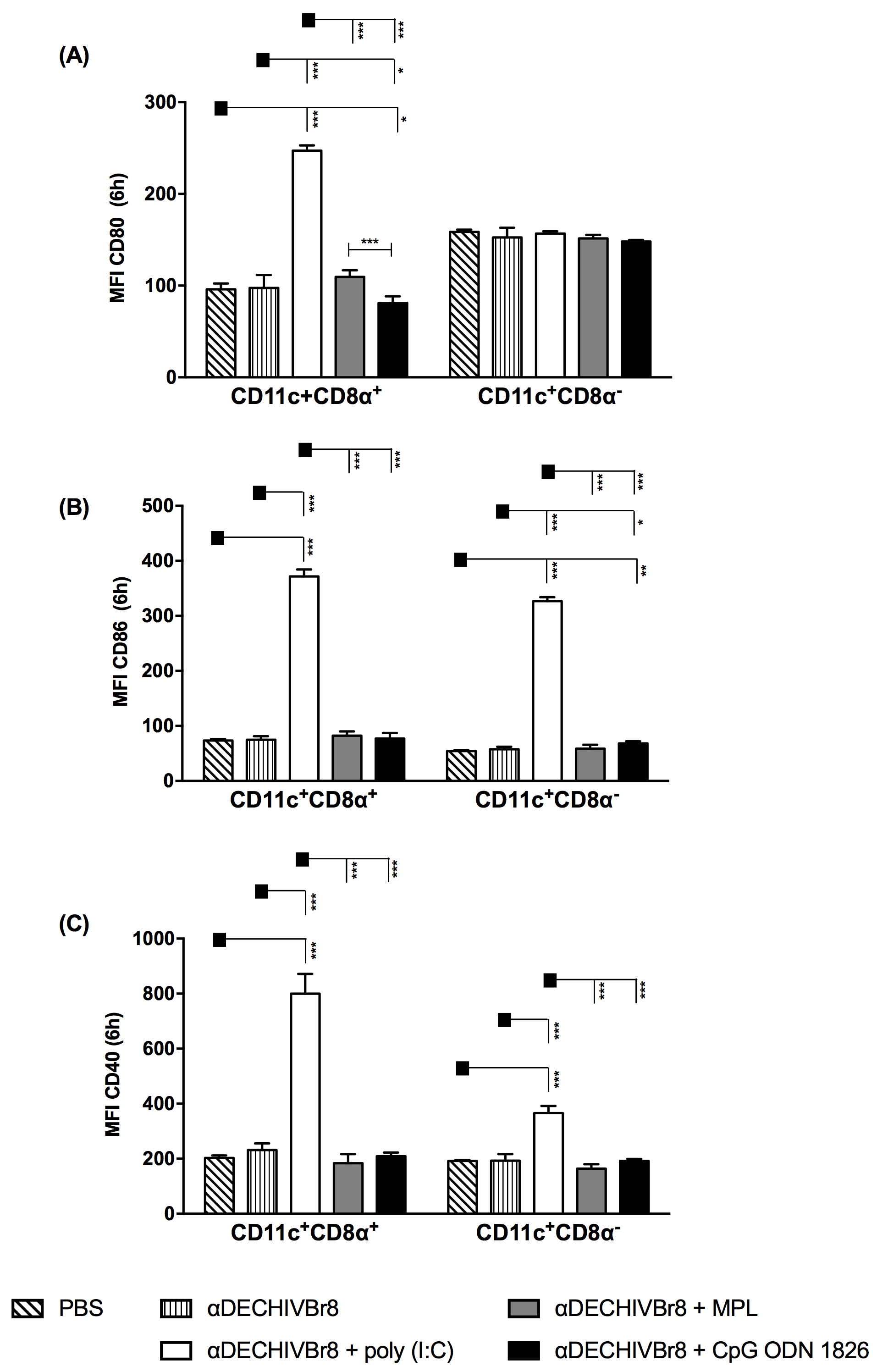
**

**Supplementary Figure 4. DC targeting using different adjuvants induces early activation in splenic DCs subsets.** BALB/c mice (n=5) were injected with 4μg of αDECHIVBr8 combined with poly (I:C), MPL or CpG ODN 1826. Control groups received αDECHIVBr8 only or PBS. After 6 hours the splenocytes were labeled with fluorescent antibodies and gated as described in Sup Fig 3. The median fluorescence intensity (MFI) of CD80 **(A)**, CD86 **(B)** and CD40 **(C)** was determined in each DC subset. Data were analyzed by two-way ANOVA followed by Bonferroni post hoc test*.* *p< 0.05, **p<0.01; ***p<0.001. Data represent mean ± SD and are representative of 3 independent experiments.
